# Supplementary figures and images for: Correlation of Matrisome-Associatted Gene Expressions with LOX Family Members in Astrocytomas Stratified by IDH Mutation Status
Source: Int J Mol Sci. 2022 Aug 23;23(17):9507. doi: 10.3390/ijms23179507 (PMC9455728; doi:10.3390/ijms23179507)

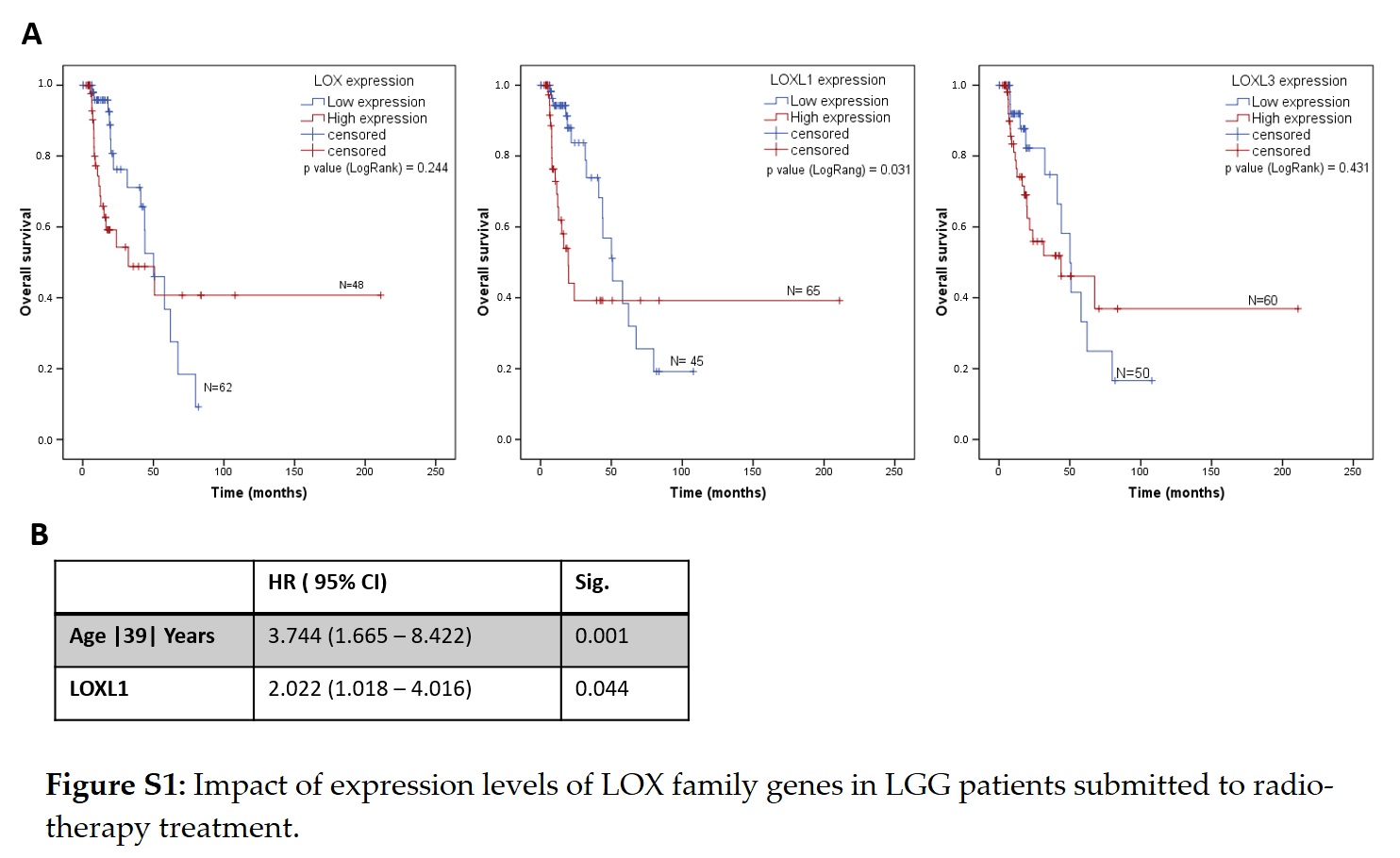

Supplement: Supplementary file 1 [file ijms-23-09507-s001.zip › Figure S1.jpg]
